# The prediction of the allergenicity food proteins by combining an in vitro and an in vivo model

**DOI:** 10.1186/2045-7022-5-S3-P153

**Published:** 2015-03-30

**Authors:** Jolanda Van Bilsen, Marylène De Zeeuw-Brouwer, Manon Van Roest, Govardus De Jong, Raymond Pieters, Geert Houben, Joost Smit

**Affiliations:** 1 TNO, Utrecht, The Netherlands; 2 TNO, Zeist, The Netherlands; 3 Institute for Risk Assessment Sciences, Utrecht, The Netherlands

## 

There is a growing need to develop methods that characterize the allergenic potential of novel and modified food proteins. To date there are no validated in vitro and in vivo models and non-validated assays showed false positive results for non/low-allergenic proteins. Previously, we showed that the combination of a DC-T cell assay and a murine in vivo model was able to distinguish strong allergenic proteins (Ara h1, b-Lactoglobulin (b-LG)) from low/non-allergenic proteins (soy lipoxygenase (SL), gelatin). Here we extended the panel of test proteins (beef tropomyosin (BT), rubisco, patatin, BSA) to investigate the specificity of the assays further. Moreover we tested the sensitivity of the in vivo model by testing whether glycosylation of WPC would result in an increased allergenicity.

*DC-T assay:* A CD4+ T cell pool, containing protein-specific T cells was obtained from protein/alum-immunized C3H/HeOuJ mice. The CD4+ T cells were incubated for 72 hrs with protein-pulsed bone marrow derived dendritic cells (DC). Hereafter,IL-5, IL-10, IL-13 and IFN- y production was analyzed.

*Mouse model:* C3H/HeOuJ mice were sensitized by intra-gastric administration of protein with cholera toxin on days 0 and 7. On day 16, mice were challenged intra-gastrically with protein. Serum was analyzed for protein-specific antibodies and mouse mast cell protease-1 (mMCP-1).

Ara h1, b-LG, BSA-, rubisco- and patatin-pulsed but not SL-, gelatin- or BT-pulsed DC induced the production of IL-5, IL-10 and IL-13 from T cells. Thepatatin- and rubisco-induced cytokine production was accompanied by IFN-y induction and likely due to LPS contamination, indicating the LPS-sensitivity of the DC-T assay. In vivo, all allergens induced IgE and mMCP-1 release, whereas the low/non-allergens did not. Rubisco and BT were not tested in vivo. The degree of glycosylation of WPC resulted in similar increase in IgE and mMCP-1 release.

Concluding, both the in vitro DC-T cell assay and the in vivo mouse model were able to distinguish known allergens from low/non-allergenic proteins. This may be a promising testing strategy for new (possibly allergenic) proteins in the diet which will be the focus of future research.

